# Threat of Shock and Aversive Inhibition: Induced Anxiety Modulates Pavlovian-Instrumental Interactions

**DOI:** 10.1037/xge0000363

**Published:** 2017-09-14

**Authors:** Anahit Mkrtchian, Jonathan P. Roiser, Oliver J. Robinson

**Affiliations:** 1 Institute of Cognitive Neuroscience, University College London

**Keywords:** threat of shock, Pavlovian-instrumental interactions, anxiety, behavioral inhibition, go/no-go

## Abstract

Anxiety can be an adaptive response to potentially threatening situations. However, if experienced in inappropriate contexts, it can also lead to pathological and maladaptive anxiety disorders. Experimentally, anxiety can be induced in healthy individuals using the threat of shock (ToS) paradigm. Accumulating work with this paradigm suggests that anxiety promotes harm–avoidant mechanisms through enhanced inhibitory control. However, the specific cognitive mechanisms underlying anxiety-linked inhibitory control are unclear. Critically, behavioral inhibition can arise from at least 2 interacting valuation systems: instrumental (a goal-directed system) and Pavlovian (a “hardwired” reflexive system). The present study (*N* = 62) replicated a study showing improved response inhibition under ToS in healthy participants, and additionally examined the impact of ToS on aversive and appetitive Pavlovian-instrumental interactions in a reinforced go/no-go task. When Pavlovian and instrumental systems were in conflict, ToS increased inhibition to aversive events, while leaving appetitive interactions unperturbed. We argue that anxiety promotes avoidant behavior in potentially harmful situations by potentiating aversive Pavlovian reactions (i.e., promoting avoidance in the face of threats). Critically, such a mechanism would drive adaptive harm–avoidant behavior in threatening situations where Pavlovian and instrumental processes are aligned, but at the same time, result in maladaptive behaviors when misaligned and where instrumental control would be advantageous. This has important implications for our understanding of the mechanisms that underlie pathological anxiety.

Anxiety disorders constitute a leading global disease burden (Baxter, Vos, Scott, Ferrari, & Whiteford, 2014), but their neurocognitive underpinnings are poorly understood. Indeed, we have relatively little understanding of the effects of *adaptive* anxiety in healthy individuals. This is important, as the mechanisms underlying adaptive anxiety are thought to form the basis of pathological anxiety (Grupe & Nitschke, 2013; Robinson, Vytal, Cornwell, & Grillon, 2013).

Adaptive anxiety can be explored in healthy individuals using the threat of shock (ToS) paradigm (Robinson, Vytal, et al., 2013; Schmitz & Grillon, 2012). This paradigm reliably increases general response inhibition (Aylward & Robinson, 2017; Grillon, Robinson, Mathur, & Ernst, 2016; Robinson, Krimsky, & Grillon, 2013; Torrisi et al., 2016) and facilitates aversive processing (Robinson, Vytal, et al., 2013). These observations, together with avoidance behaviors in pathological anxiety (Craske et al., 2009), support the hypothesis that adaptive anxiety promotes harm–avoidant behavior through aversive-linked inhibitory control.

Aversive-linked inhibition may, however, be driven by at least two separate but parallel valuation systems: Pavlovian and instrumental (Dickinson & Balleine, 2002). The Pavlovian system reflects reflexive, evolutionary-appropriate behavioral patterns to outcomes (rewards/punishments) or stimuli associated with these outcomes through classical conditioning. The general prepotent response of the Pavlovian system toward potential rewards is response vigor (approach), producing a Pavlovian “go” bias in the face of rewards. Pavlovian responses in the face of potentially negative outcomes are generally associated with avoidance or inhibitory responses (McNaughton & Corr, 2004).^1^ Action and valence are therefore thought to be intrinsically coupled in the Pavlovian system (i.e., instinctively producing invigorating actions toward rewards/inhibitory responses in the face of punishments). It is believed that these behavioral patterns were promoted by evolution due to their advantage of increasing survival. They are therefore optimal and efficient in many environments, but fail to adapt to situations requiring different actions to outcomes than the preprogrammed patterns (Rangel, Camerer, & Montague, 2008).

Instrumental behaviors (i.e., instrumental conditioning), by contrast, are flexible behaviors based on learning the appropriate actions (approach or inhibition) to maximize rewards and minimize punishments. Action and valence are therefore independent from one another in the instrumental system which generates the optimal actions to optimize outcomes in a situation specific manner.

Behavior is ultimately guided by an interaction between Pavlovian and instrumental processes (Dayan, Niv, Seymour, & Daw, 2006; Guitart-Masip, Duzel, Dolan, & Dayan, 2014; Guitart-Masip et al., 2012; Rigoli, Pavone, & Pezzulo, 2012; Talmi, Seymour, Dayan, & Dolan, 2008) such that performance is facilitated when these systems are aligned by promoting the same actions (e.g., both promote go actions), but impaired when in conflict by producing opposite actions (e.g., Pavlovian system generates go actions while instrumental promotes no-go actions). For example, when there is a perceived benefit to withholding responses to rewards for the promise of a better outcome in the future (e.g., dieting) the instrumental system has to override the reflexive Pavlovian bias to approach rewards. Importantly, most tasks (e.g., O’Doherty et al., 2004) omit conditions that place these systems in conflict (i.e., “go to avoid punishment” and “no-go to obtain reward”), meaning that teasing these processes apart has not been possible in prior studies.

Enhanced aversive Pavlovian processes have been observed in anxiety disorders (Duits et al., 2015; Lissek et al., 2005), and induced anxiety in healthy individuals shifts the balance away from deliberative toward more automatic behaviors (Otto, Raio, Chiang, Phelps, & Daw, 2013; Schwabe & Wolf, 2011). ToS has also been shown to enhance the neural substrates of aversive but not appetitive Pavlovian conditioning (Robinson, Overstreet, Charney, Vytal, & Grillon, 2013), but the influence of Pavlovian processes over instrumental responses during ToS is yet to be explored. This question is particularly important in light of recent research suggesting that aberrant Pavlovian-instrumental interactions, especially during conflict, constitute a core mechanism underlying affective disorders, driven mainly by anomalous Pavlovian rather than instrumental processes (Boureau & Dayan, 2011; Dayan & Huys, 2008; Huys et al., 2011, 2012; Huys et al., 2016; Huys, Guitart-Masip, Dolan, & Dayan, 2015; Itzhak, Perez-Lanza, & Liddie, 2014).

To address this question, we examined the behavior of healthy volunteers during instructed Pavlovian-instrumental interactions under threatening and safe conditions. We first replicated the effect of ToS on a nonvalenced inhibitory control task (Aylward & Robinson, 2017; Grillon et al., 2016; Robinson, Krimsky, et al., 2013; Torrisi et al., 2016), demonstrating (as predicted) improved inhibition under threat as a positive control. We next explored the effect of ToS on a reinforced go/no-go task where action (go/no-go) and valence (reward/punishment) were varied orthogonally to create conditions where the Pavlovian and instrumental systems were either aligned (“go to obtain reward”; “no-go to avoid punishment”) or in conflict (“go to avoid punishment”; “no-go to obtain reward”). The main outcome measure assessing Pavlovian-instrumental interactions in this task is response latencies (Crockett, Clark, & Robbins, 2009), such that faster responses mark go actions and slowing of responses indicate inhibition (no-go) of actions. Previous studies have demonstrated that performance on such tasks is altered when the Pavlovian and instrumental systems conflict as demonstrated by either reduced accuracy or altered response times (e.g., Crockett et al., 2009; Guitart-Masip et al., 2012). Based on the theoretical view that anxiety promotes aversive inhibitory Pavlovian processing (Dayan & Huys, 2008), we hypothesized that the Pavlovian no-go bias in the face of potential losses would be amplified by ToS. Specifically, we predicted that ToS would result in increased inhibition, selective to punishment, when the Pavlovian and instrumental systems were in conflict.

## Method

### Participants

Sixty-two healthy participants (39 females; age range = 18–57; *M*_age_ = 27.16, *SD* = 7.83) were recruited from the University College London (UCL) Institute of Cognitive Neuroscience Subject Database. Sample size was determined by an a priori power analysis in G*Power (Faul, Erdfelder, Lang, & Buchner, 2007). The power analysis was based on the main finding from the reinforced go/no-go task showing that participants are significantly slower to respond in the punished conditions relative to the rewarded conditions, with a Cohen’s *d*_*z*_ (within-subjects) effect size of 0.487 (Crockett et al., 2009). Detecting an effect size of this magnitude using a paired *t* test requires 57 participants at the 0.05 alpha level (two-tailed) with 95% power. The present study recruited 62 participants to allow for a small number of unusable data sets.

Due to a recording fault during the sustained attention to response task (SART), one female participant was excluded, resulting in 61 participants in the SART. Participants reported no history of psychiatric, neurological or substance use disorders and no pacemaker implantation. Participants provided written informed consent and were reimbursed £7.50/hr for participation. To incentivize performance, participants were also informed that they could receive additional financial compensation based on task performance. The study obtained ethical approval from the UCL Research Ethics Committee (Project ID Number: 1764/001) and was conducted in accordance with the Declaration of Helsinki. Data and materials for the tasks are freely available for download (https://figshare.com/articles/SART_script/3443093 and https://dx.doi.org/10.6084/m9.figshare.c.3291299.v1).

### Procedure

The ToS procedure is identical to Mkrtchian, Aylward, Dayan, Roiser, and Robinson (2017). Anxiety was induced with the ToS paradigm where unpredictable electric shocks were delivered with two electrodes attached to the nondominant wrist using a Digitimer DS5 Constant Current Stimulator (Digitimer Ltd, Welwyn Garden City, U.K.). A highly unpleasant but not painful (Schmitz & Grillon, 2012) subjective shock level was established using a shock work-up procedure prior to testing. No more than five (to avoid habituation) shocks with a gradually increasing shock level were administered. Participants rated each shock on a scale from 1 (*barely felt it*) to 5 (*unbearable*) to reach a shock level of 4. The reinforced go/no-go task was programmed in Psychtoolbox (http://psychtoolbox.org) and the SART in Cogent (Wellcome Trust Centre for Neuroimaging and Institute of Cognitive Neuroscience, UCL, London, U.K.) using MATLAB (Release 2014a, The MathWorks, Inc., Natick, MA, United States).

Both tasks were presented on a laptop and administered under alternating safe and threat blocks. During the safe block, the background color was blue and the block was preceded by a 4000ms message stating, “You are now safe from shock.” During the threat block, the background color was red and the message, “Warning! You are now at risk of shock” was presented for 4,000 ms at the beginning. Participants were told that they might receive a shock only during the threat condition but that the shocks were not dependent on their performance. At the end of each experimental task, participants retrospectively rated how anxious, stressed and afraid they felt during the safe and threat conditions on a scale from 1 (*not at all*) to 10 (*very much so*). Numerous previous studies have implemented this questionnaire to assess effectiveness of the threat condition (Robinson, Vytal, et al., 2013). The reinforced go/no-go task and SART were administered together with a third task (the third task was part of a larger study including a patient group and are published separately: Mkrtchian et al., 2017). All experimental tasks were administered in a counterbalanced order across participants.

#### SART

The SART (nonvalenced inhibition task) was programmed in Cogent using MATLAB. Participants were presented with frequent “go” stimuli (“=”), during which they had to press spacebar and infrequent “no-go” stimuli (“O”), when they were required to withhold a response. The stimuli were presented in a random order, for 250 ms with a 1,750 ms intertrial interval (ITI). In each block the go-trials occurred 47 or 48 times while the no-go trials were presented four or five times: 190 go and 18 no-go trials in total across all safe or threat blocks. The task was run in eight blocks, alternating threat and safe conditions (Robinson, Krimsky, et al., 2013). The order of the safe and threat blocks was counterbalanced across participants. The task lasted approximately 18 min with one shock delivered in the first, second and last threat block. Participants were asked to respond as quickly and accurately as possible.

#### Reinforced go/no-go task

**Figure 1 fig1:**
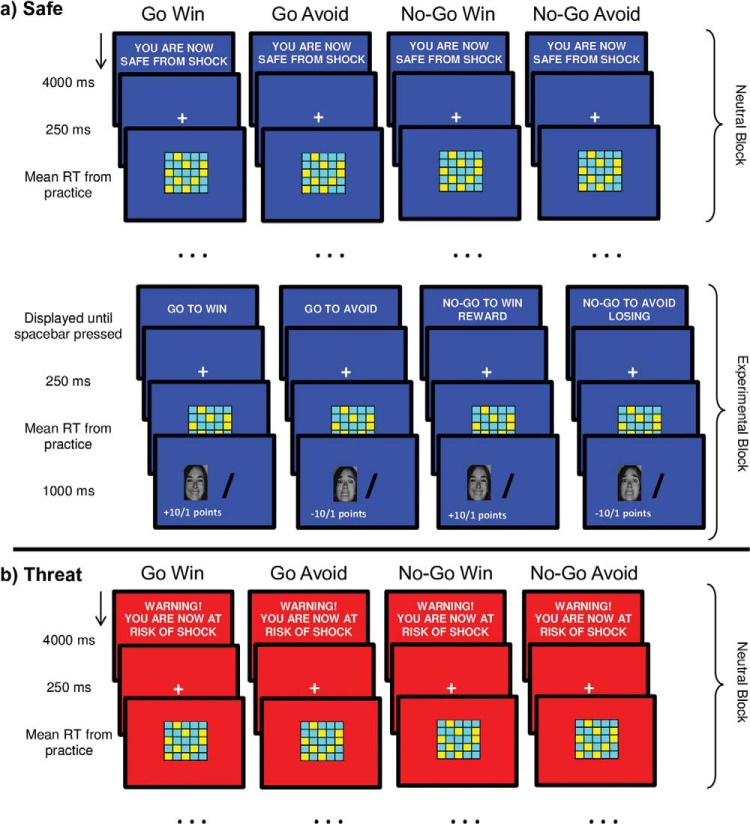
Trial sequence for all the action–valence (A-V) conditions in the reinforced go/no-go task. Each A-V condition began with a neutral block (36 trials) to allow reaction times (RTs) to equilibrate, followed by one of the four A-V experimental conditions (36 trials). Displayed are the trial sequence for each A-V condition under (a) safe and (b) threat. The safe and threat blocks were presented in alternating order, counterbalanced across participants. See the online article for the color version of this figure.

The reinforced go/no-go task was programmed in Psychtoolbox using MATLAB. The task was adapted from Crockett et al. (2009), which is based on a signal-detection paradigm. Participants were asked on each trial to determine if a target tile color was in the majority or minority in a checkerboard. They were required to press the spacebar (go response) if the target color (blue or yellow, counterbalanced across participants) was in the majority (go trial) and to withhold (no-go response) a keypress if the target color was in the minority (no-go trial; Figure 1). Thus, the signal in this task is the color that is in the majority (i.e., go trials). As in Crockett et al. (2009), go and no-go trials were equally divided between easy (16:9 blue/yellow tile ratio and vice versa) and difficult (13:12 blue/yellow tile ratio and vice versa) checkerboards.[Fig-anchor fig1]

**Figure 2 fig2:**
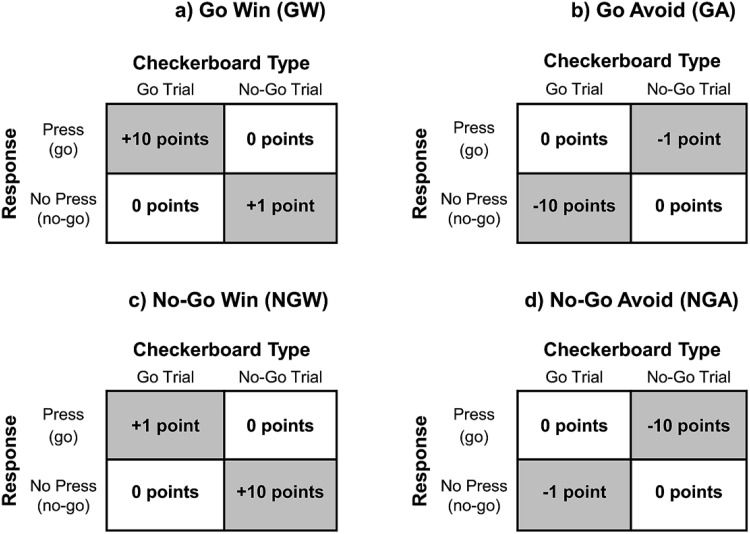
The action–outcome reinforcement schedules for each action–valence task condition. (a) In the go win (GW) condition, actions were biased toward go, by rewarding correct go responses more strongly than correct no-go responses; incorrect responses were not rewarded. (b) In the go avoid (GA) condition actions were biased toward go, by punishing incorrect no-go responses more harshly than incorrect go responses; correct responses were not punished. (c) In the no-go win (NGW) condition actions were biased toward no-go, by rewarding correct no-go responses more strongly than correct go responses; incorrect responses were not rewarded. (d) In the no-go avoid (NGA) condition actions were biased toward no-go, by punishing incorrect go responses more harshly than incorrect no-go responses; correct responses were not punished.

The task comprised four action–valence (A-V) conditions where action (go/no-go) was crossed with valence (reward/punishment): go to win reward (GW), go to avoid punishment (GA), no-go to win reward (NGW), and no-go to avoid punishment (NGA). All A-V conditions included checkerboards with both 50% go trials and 50% no-go trials, such that we were able to acquire go RTs in all four A-V conditions. Critically, to bias responses toward the different actions (go/no-go), some responses were rewarded or punished more strongly depending on the A-V condition. Specifically, responses were biased toward go in the GW condition by rewarding correct go responses more than correct no-go responses. Similarly, correct no-go responses were rewarded more than correct go responses in the NGW condition. In the GA condition, responses were biased toward go by punishing incorrect go responses less severely than incorrect no-go responses. Finally, incorrect go responses were punished more severely than incorrect no-go responses in the NGA condition to bias responses toward no-go in this condition (see Figure 2). Large rewards received 10 points and a happy face; small rewards earned 1 point and a happy face. For large punishments, participants lost 10 points and received a fearful face; for small punishments, they lost 1 point and received a fearful face. Faces were chosen from the Ekman stimuli (Ekman & Friesen, 1976) for consistency with our prior work (Robinson, Vytal, et al., 2013) with stimulus gender counterbalanced across participants. The outcome was presented for 1,000 ms with a 250-ms ITI.[Fig-anchor fig2]

The task began with two practice blocks, both without the influence of the threat manipulation. The first practice block began with 48 neutral (without outcomes) practice trials. Participants were asked to respond to the checkerboards as quickly and accurately as possible. The stimuli were presented for 2,000 ms with a 250-ms ITI. The second practice block comprised of guided practice blocks for each A-V condition (order randomized across participants), containing four trials each, allowing participants to learn the action–outcome contingencies for each A-V task condition. To yoke task difficulty with respect to individual differences in reaction time (RT), the stimulus duration for the main task was set as the mean RT of the correct responses from the first practice block.

The main task had eight blocks in total (four threat, four safe), with the safe/threat block order counterbalanced across participants. The A-V conditions were presented in blocks and occurred twice (once under threat and once under safe), with safe/threat order randomized across participants (see Figure 1). Each block started with a neutral condition (36 nonreinforced trials), to allow RTs to return to baseline and thus avoid any carryover effects from previous reinforced blocks, followed by one out of the four A-V conditions (36 trials): GW, GA, NGW, NGA. Prior to the start of each A-V condition, participants were explicitly informed which A-V condition they were about to complete by text instructions on the screen. To begin each A-V experimental block, participants had to press the spacebar after reading the text instruction. The task lasted around 25 min with one shock delivered during task performance in the second and fourth threat blocks.

### Data Analysis

All data were analyzed in SPSS version 22 (IBM Corp, Armonk, NY) and inspected for deviations from normality assumptions prior to analysis (of which none were found). For all analyses, *p* < .05 was considered statistically significant. For all paired *t* test analyses, Cohen’s *d*_z_ effect size (within-subjects) was calculated (Lakens, 2013). The index of variation in figures was calculated according to the formula (Equation 1) by Loftus and Masson (1994) as standard error of the mean (SEM) is not appropriate error information for within-subjects designs.

$${\text{SEM}}_{\text{within}}\;=\;\text{SQRT(MSE}/\text{n),}$$ 1

where *MSE* represents the mean squared error of the relevant main effect from the repeated-measures analysis and *n* represents the number of participants. The SEM_within_ captures the within-subjects variance only (changes in scores from safe to threat conditions within each participant) by removing between-subjects variance (differences between participants) and is therefore an appropriate method to illustrate graphically the differences in means in within-subjects designs.

Frequentist statistics were supplemented with Bayesian statistics to quantify the confidence in the main null effects. Bayesian analyses were performed in JASP Version 0.8.1.1 using the default prior (Love et al., 2014; Morey & Rouder, 2015; Rouder, Morey, Speckman, & Province, 2012). Bayesian statistics were used to obtain Bayes factors (BF_10_) for the model of interest, relative to the null model (main effect of participants). To facilitate interpretation of the magnitude difference between models (BF_10_ of model of interest divided by the BF_10_ of the comparison model), a model 1–3 times better than the comparison model was considered “anecdotal,” 3–10 was “substantial,” 10–30 was “strong,” 30–100 was “very strong,” and >100 was “decisive” (Jeffreys, 1998).

#### Anxiety manipulation check

Paired *t* tests were used to analyze the retrospective ratings of anxiety, stress and fear during the threat and safe conditions.

#### SART

Percent correct scores were analyzed on no-go trials during the threat and safe conditions (go accuracy across the safe and threat condition was above 97%) using a paired *t* test. RTs were analyzed with a paired *t* test (correct go trials only) between threat and safe conditions.

#### Reinforced go/no-go task

##### Primary analysis: Pavlovian-instrumental interactions

As the present study was based on the original study by Crockett and colleagues (2009), our Pavlovian-instrumental analysis aims to replicate the original approach as closely as possible. Pavlovian-instrumental interactions were analyzed by extracting RTs for correct go trials only (i.e., a go response on a go trial) for all A-V conditions in threat and safe blocks. In line with Crockett et al. (2009), RTs were collapsed across easy and difficult trials. RTs were normalized within subject (using the mean and standard deviation of the first practice block) to assess the influence of each A-V condition (GW, GA, NGW, NGA) on RTs. Prior to analysis, we reasoned that the first practice block (48 trials) would provide the most accurate and appropriate measure of baseline RTs in our study. This is in contrast to Crockett et al. (2009), where the first neutral 36 trials (without outcomes) from the main task were used as baseline. This is because in the present study the first 36 trials in each A-V condition alternated between threat and safe across participants due to the within-session threat manipulation. The first practice was, by contrast, experienced prior to the ToS manipulation. The normalized RTs were analyzed with a repeated-measures analysis of variance (ANOVA) with threat (threat, safe), action bias (go, no-go) and valence (reward, punishment) as within-subjects factors. More negative z-score values indicate faster go responses.

##### Secondary analyses: Go/no-go bias check

Although the reinforced go/no-go task is primarily designed to measure RT differences between the A-V conditions (Crockett et al., 2009), we also examined accuracy to assess if the go/no-go bias manipulation had worked as intended. Analyses of hit rate (HR) and false alarm rate (FAR) scores were conducted to assess performance on go and no-go trials across the different A-V conditions. Response bias from signal detection theory was also calculated (Stanislaw & Todorov, 1999). This measure reflects whether the asymmetric reward and punishment reinforcement schedules were effective in generating the expected response biases in each A-V condition. Analyses of HR scores, FAR scores and response bias were restricted to difficult trials due to ceiling performance on the easy trials (>90% accuracy, Figure S1 in the online supplemental materials).

Response bias was measured by calculating *c,* derived from signal detection theory (Stanislaw & Todorov, 1999) for each A-V experimental condition (Equation 2):

c=−0.5(z(HR)+z(FAR)), 2

where *z* represents the inverse of the cumulative Gaussian distribution, HR is the hit rate (correct go responses divided by the total number of go trials) and FAR is the false alarm rate (incorrect no-go divided by the total number of no-go trials). A negative value of *c* indicates a greater tendency toward go responses while positive values indicate a bias toward no-go responses.

The “log-linear” approach (Hautus, 1995) was used to deal with cases of 1 or 0 HRs/FARs for the response bias calculation. This involves adding 0.5 to the number of hits and the number of false alarms and 1 to the total number of go and no-go trials. HR, FAR and response bias were analyzed using repeated-measures ANOVAs with threat (threat, safe), action bias (go, no-go), and valence (reward, punishment) as within-subject factors.

## Results

### Anxiety Manipulation Check

While performing the SART, participants rated themselves significantly more anxious (*M* = 5.38, SEM = 0.31; *t*(61) = 12.82, *p* < .001, *dz* = 1.64, 95% confidence interval (CI) for the mean difference [3.06, 4.19]), stressed (*M* = 5.13, SEM = 0.31; *t*(61) = 10.61, *p* < .001, *dz* = 1.36, 95% CI for the mean difference [2.50, 3.66]), and afraid (*M* = 4.54, SEM = 0.32; *t*(61) = 11.21, *p* < .001, *dz* = 1.44, 95% CI for the mean difference [2.63, 3.77]) during threat relative to safe blocks (safe ratings of anxiety: *M* = 1.75, SEM = 0.16, stress: *M* = 2.05, SEM = 0.20, fear: *M* = 1.34, SEM = 0.11). Similarly, while performing the reinforced go/no-go task, participants rated themselves significantly more anxious (*M* = 5.06, SEM = 0.32; *t*(61) = 8.64, *p* < .001, *dz* = 1.10, 95% CI for the mean difference [2.37, 3.80]), stressed (*M* = 4.97, SEM = 0.34; *t*(61) = 8.94, *p* < .001, *dz* = 1.14, 95% CI for the mean difference [2.24, 3.53]), and afraid (*M* = 4.47, SEM = 0.32; *t*(61) = 10.17, *p* < .001, *dz* = 1.29, 95% CI for the mean difference [2.53, 3.76]) during threat relative to safe blocks (safe ratings of anxiety: *M* = 1.98, SEM = 0.21, stress: *M* = 2.08, SEM = 0.22, fear: *M* = 1.32, SEM = 0.15).

### SART

#### Threat increases no-go accuracy

**Figure 3 fig3:**
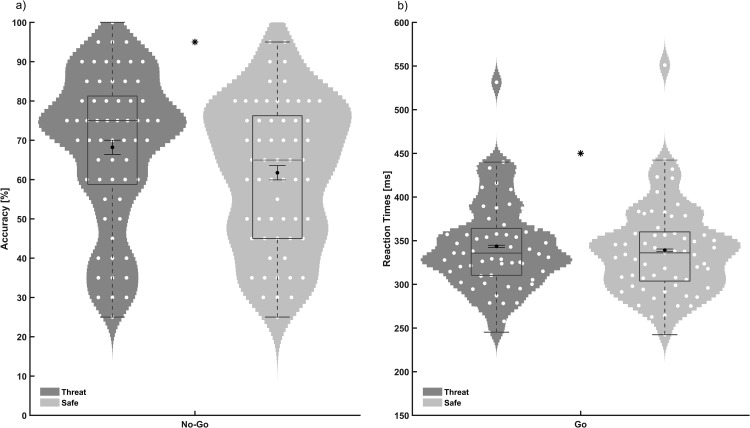
Violin and overlaid box and error bar plots of the sustained attention to response task data. (a) Accuracy on the no-go trials across threat and safe conditions. Threat significantly increased accuracy compared with safe blocks (* *p* = .001). (b) Reaction times on go trials across threat and safe conditions. Threat significantly slowed responses (* *p* = .019). Black dots represent the mean and associated error bars represent standard error of the mean for within-subjects variance.

As expected, participants performed more accurately on no-go trials under threat relative to safe conditions, *t*(1,60) = 3.57, *p* = .001, *dz* = 0.46, 95% CI for the mean difference [2.84, 10.11] (Figure 3a), replicating previous findings (Aylward & Robinson, 2017; Grillon et al., 2016; Robinson, Krimsky, et al., 2013; Torrisi et al., 2016).[Fig-anchor fig3]

#### Threat slows responses on correct go trials

Participants were significantly slower to respond correctly on go trials under threat relative to safe, *t*(60) = 2.41, *p* = .019, *dz* = 0.31, 95% CI for the mean difference [0.75, 8.03] (Figure 3b).

### Reinforced Go/No-Go Task

#### Threat Increases punishment-induced inhibition when action is biased toward go

There were significant main effects of action bias, *F*(1, 61) = 47.77, *p* < .001, η_p_^2^= 0.439, and valence, *F*(1, 61) = *5*.00, *p* = .029, η_p_^2^= 0.076, and a significant action bias by valence interaction, *F*(1, 61) = 8.38, *p* = .005, η_p_^2^= 0.121, on normalized RTs for correct go trials. Pairwise comparisons indicated that participants were significantly slower in the GA condition (*Z* scores: *M* = −1.06, SEM = 0.04; Pavlovian and instrumental in conflict) compared with the GW condition (*M* = −1.12, SEM = 0.04; Pavlovian and instrumental aligned), *F*(1, 61) = 14.19, *p* < .001, η_p_^2^ = 0.189, 95% CI for the mean difference [0.03, 0.09]. However, there was no significant difference between the NGW and NGA conditions, *F*(1, 61) = 0.055, *p* = .82, η_p_^2^ = 0.001, 95% CI for the mean difference [−0.04, 0.03]. This interaction thus replicates the predicted pattern of Pavlovian and instrumental conflict in the go conditions (e.g., Guitart-Masip et al., 2014).

**Figure 4 fig4:**
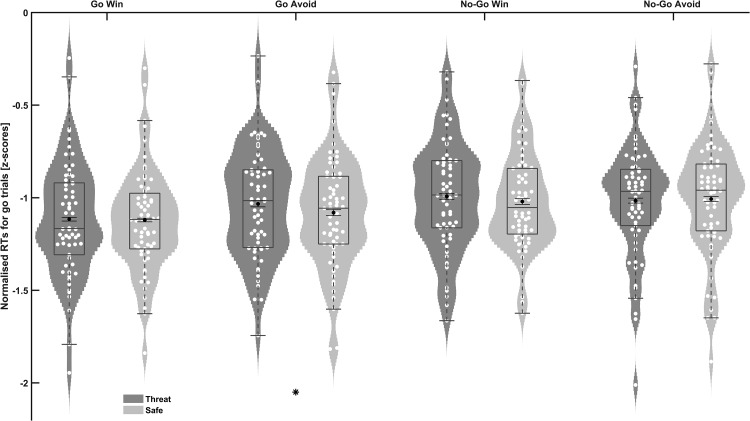
Violin plots with overlaid box and error bar plots showing normalized RTs (standardized against the practice baseline) for correct go trials in each action–valence condition under threat and safe conditions. Less negative values indicate slower responses. Threat significantly slowed responses during go to avoid punishment compared with safe blocks (* *p* = .04). This indicates that threat selectively potentiates inhibition of actions in the face of punishment when the Pavlovian and instrumental systems are in conflict, by increasing reliance on aversive Pavlovian biases. Black dots depict the mean and associated error bars represent standard error of the mean for the within-subjects variance.

Importantly, these effects were qualified by a significant three-way interaction between threat, action bias and valence, *F*(1, 61) = 4.83, *p* = .032, η_p_^2^ = 0.073. This interaction was driven by a significant action bias by valence interaction under threat, *F*(1, 61) = 10.39, *p* = .002, η_p_^2^ = 0.146, but not under safe, *F*(1, 61) = 1.06, *p* = .309, η_p_^2^= 0.017. Under threat, participants were significantly slower to make a correct response to avoid punishment (GA) than to obtain reward (GW; i.e., when the action was biased toward go), *F*(1, 61) = 13.32, *p* = .001, η_p_^2^= 0.179, but not when actions were biased toward no-go (no difference between NGW and NGA), *F*(1, 61) = 1.11, *p* = .30, η_p_^2^= 0.018. Importantly, participants were significantly slower under threat than safe for the GA condition, *F*(1, 61) = 4.39, *p* = .04, *dz* = 0.27, 95% CI for the mean difference [0.002, 0.093] (Figure 4), but there was no significant difference between threat and safe for the other three conditions (GW, NGW, NGA all *p* > .05; Figure 4). Indeed, Bayes factor analysis showed that the null model was substantially better than the threat model for GW (BF_10_ = 0.15), NGW (BF_10_ = 0.29) and NGA (BF_10_ = 0.16). Post hoc paired *t* tests revealed no significant differences between GA under threat with NGA under threat or safe (all *t*s < 2, all *p*s > 0.2).[Fig-anchor fig4]

In summary, ToS only enhanced inhibition of actions (driven by Pavlovian aversive processing) in the face of potential punishment when the Pavlovian and instrumental systems were in conflict. In other words, acute anxiety promoted increased reliance on Pavlovian biases in aversive but not appetitive conflict contexts.

#### Go/no-go bias check

##### Response bias

The effectiveness of the reward and punishment reinforcements were assessed with response bias, *c*, with negative values indicating a greater propensity toward go responses and positive values toward no-go responses. There was only a significant main effect of action bias, *F*(1, 61) = 41.43, *p* < .001, η_p_^2^= 0.404, 95% CI for the mean difference [0.24, 0.46]. Participants were thus biased toward go responses in the go conditions (*M* = −0.25, SEM = 0.05) and toward no-go in the no-go conditions (*M* = 0.10, SEM = 0.05). This indicates that the task payoff schedule for each condition (bias actions toward go vs. no-go by rewarding/punishing actions differentially) worked as intended. All other main effects and interactions were nonsignificant (all *F*s < 3, all *p*s > 0.1), suggesting that threat does not specifically affect *sensitivity* to punished or rewarded outcomes. Indeed, according to Bayes factor analysis, all models that included threat as a factor were at least 17 times worse than the action bias model, providing strong evidence against an effect of threat.

##### Hits and false alarms

Consistent with the pattern of response bias, there was a main effect of action bias, *F*(1, 61) = 41.93, *p* < .001, η_p_^2^ = 0.407, 95% CI for the mean difference [0.10, 0.19], on HR. As expected, participants made more hits in go (*M* = 71.33%, SEM = 1.76%) relative to no-go (*M* = 57.12%, SEM = 1.97%) conditions. No other main effect or interaction was significant (all *F*s < 3, all *p*s > 0.1). All models that included threat as a factor were at least 36 times worse than the action bias model, providing very strong evidence against an effect of threat. There was also a significant main effect of action bias on FAR, *F*(1, 61) = 26.67, *p* < .001, η_p_^2^ = 0.304, 95% CI for the mean difference [0.07, 0.15]. Participants thus committed more commission errors on no-go trials when the action was biased toward go (*M* = 46.73%, SEM = 1.95%) than when the payoff schedule biased participants’ actions toward no-go (*M* = 35.84%, SEM = 2.00%), replicating the results of Crockett et al. (2009). This effect was also not modulated by threat, nor was any other main effect or interaction significant (all *F*s < 3, all *p*s > 0.1). Similarly, Bayes factor analysis showed that all models that included threat as a factor were at least 21 times worse than the action bias model, providing strong evidence against an effect of threat.

## Discussion

In this study we replicate prior work demonstrating increased response inhibition under ToS, while at the same time extending these findings to encompass inhibition in aversive and appetitive contexts. We argue that threat-potentiated inhibition is selective for go responses associated with potentially negative outcomes. This is consistent with the proposition that adaptive anxiety potentiates harm–avoidant behavior (Robinson, Vytal, et al., 2013) and, importantly, could plausibly be driven by an aversive Pavlovian bias overriding instrumental behavioral control. In other words, these findings support the novel proposition that one cognitive consequence of anxiety is an increased reliance on aversive, but not appetitive, Pavlovian control over behavior.

Our results suggest that ToS is involved in facilitating the coupling between response inhibition and punishment predictions in motivational behavior. This is evidenced by slowed responses specifically when go actions were required to avoid punishments during threat. Overall performance in the GA condition was slowed relative to the condition when Pavlovian and instrumental behaviors cooperate (i.e., GW). This indicates that the task worked as intended (at least when actions were biased toward go), as performance was altered when Pavlovian and instrumental controllers favored opposite behaviors (Crockett, Clark, Apergis-Schoute, Morein-Zamir, & Robbins, 2012; Crockett et al., 2009; Guitart-Masip et al., 2014; Guitart-Masip et al., 2012). Critically, however, under ToS, this slowing was exacerbated, inducing greater inhibitory responses. We argue that this slowing indicates that ToS promotes greater reliance on Pavlovian inhibitory biases during potentially punishing events, effectively abolishing the influence of instrumental control during a threatening context. Importantly, threat did not modulate performance during any of the appetitive conditions, which suggests that anxiety might only promote Pavlovian biases in the face of potential punishments.

This task also enables the separation of response inhibition and valence-specific behavioral responses. Previous research has consistently found that ToS impacts response inhibition (Aylward & Robinson, 2017; Grillon et al., 2016; Robinson, Krimsky, et al., 2013; Torrisi et al., 2016) as well as in aversive processes (Grillon & Charney, 2011; Robinson, Charney, Overstreet, Vytal, & Grillon, 2012; Robinson, Letkiewicz, Overstreet, Ernst, & Grillon, 2011). However, the commission error rates and the response bias measure indicate that neither response inhibition nor punishment signaling were modulated by threat *independently* in this task. As such, these data further suggest that anxiety in motivational behavior is particularly important for a unitary aversive-linked inhibition process. Thus, in addition to the accumulating evidence of anxiety-potentiated “neutral” response inhibition (Aylward & Robinson, 2017; Grillon et al., 2016; Robinson, Krimsky, et al., 2013; Torrisi et al., 2016), we show that anxiety specifically promotes response inhibition in a situation where the prepotent response is inhibition (i.e., when the context is specifically aversive). This suggests that ToS, by virtue of being a global aversive context, may promote a “generic” bias toward inhibition on the SART and other tasks because inhibition is the prepotent response to aversive contexts (Aylward & Robinson, 2017; de Berker et al., 2016; Grillon et al., 2016; Robinson, Krimsky, et al., 2013; Torrisi et al., 2016).

This hypothesis is consistent with theories of how adaptive anxiety impacts cognition (Robinson, Vytal, et al., 2013). Specifically, when something is potentially harmful, anxiety promotes behavior that will avoid interaction with it, at the potential detriment to goal-directed behavior. Instrumental behavior is particularly disrupted when anxiety promotes behavior that is task-incongruent (Robinson, Vytal, et al., 2013), as is the case in the go to avoid losing condition. Indeed, in the GA condition, ToS leads to a maladaptive strategy that could potentially lead to more harm in this specific context, where avoidance is promoted yet the optimal strategy should be invigoration. This coupling of actions (go to avoid punishment) is actually rare in nature (at least to distal threats; McNaughton & Corr, 2004; Rangel et al., 2008) which means that relying on Pavlovian biases likely remains adaptive on average. Nevertheless, these findings provide a possible mechanism by which maladaptive behavior may arise in anxiety disorders, where this “hard-wired” Pavlovian response can overrule more immediate “correct” behaviors. Critically, in pathological anxiety, this Pavlovian override may occur even in the absence of experimentally induced threat (Mkrtchian et al., 2017; Robinson et al., 2014).

One of the advantages of the current task is that it allows the quantification of Pavlovian-instrumental interactions with response latencies. RTs may be more sensitive to modulation than choice responses as choice provides binomial outcomes while RTs provide continuous outcomes, allowing for greater variability. This may be particularly important when investigating healthy participants. It may be the case that the mechanisms of anxiety are similar in healthy and patient populations but that the effects are exacerbated in anxiety disorders, resulting in maladaptive behaviors in the latter but not the former population (Robinson, Vytal, et al., 2013). Interestingly, we have shown that patients with mood and anxiety disorders but not healthy participants, exhibit an increased reliance on avoidance Pavlovian biases under threat in a conceptually similar task that measures choice outcomes (Mkrtchian et al., 2017). It might therefore be the case that the effect of ToS on Pavlovian-instrumental interactions is subtle and only emerges with RTs in healthy participants. Indeed, this is in line with current theoretical approaches to psychiatry that view mood disorders on a continuous rather than on a dichotomous scale (Cuthbert & Insel, 2013; Robinson et al., 2014). Effects may be subtle, and may not affect performance (i.e., winning or losing points) in healthy participants but could disrupt performance in patients. This may also explain why our choice measures (response bias, HR, and FAR) are not modulated by threat. Indeed, the original study using this task only found an effect of tryptophan depletion on the RTs on this task and not on the choice measures (Crockett et al., 2009). To clarify this however, future studies should aim to investigate performance on the present reinforced go/no-go task under ToS with patient populations as well.

Prior neuroimaging work show that ToS enhances punishment but not reward prediction error signals in the striatum during the processing of aversive Pavlovian cues-outcome associations (Robinson, Overstreet, et al., 2013). Importantly, ToS only modulates activity in the ventral and not dorsal striatum. The ventral striatum, it has been argued, underlies Pavlovian signals, while the dorsal is associated with instrumental processes (Corbit, Muir, & Balleine, 2001). Furthermore, threat-potentiated inhibition on the SART is *also* associated with increased activity in the striatum (Torrisi et al., 2016). As such, ToS might specifically affect aversive Pavlovian but not instrumental striatal circuitry. Future work should therefore explore if striatal-driven aversive Pavlovian biases may override instrumental-driven neural circuitry under ToS.

As a potential caveat, it should be noted that it is possible that ToS influences motivational behavior by impairing instrumental control rather than by promoting aversive Pavlovian control. However, we believe that this is unlikely for a number of reasons. First, the present results are consistent with the previously discussed studies indicating that anxiety affects Pavlovian mechanisms (Duits et al., 2015; Lissek et al., 2005; Robinson, Overstreet, et al., 2013). Strikingly, a recent study demonstrated that avoidance behavior in mood and anxiety disorders is driven by aversive Pavlovian biases as tested in a similar Pavlovian-instrumental task (Mkrtchian et al., 2017). Second, in contrast to instrumental behaviors, aversive Pavlovian biases are evolutionary adaptive and rapid mechanisms evolved to avoid potential harm and increase survival (Dickinson & Balleine, 2002). They may therefore have a particularly important role in adaptive anxiety, which itself is likely an evolutionary adaptive mechanism. Thus from an evolutionary standpoint, it would make sense for anxiety, a state that accompanies potentially threatening situations, to potentiate a mechanism programmed to avoid harm, as opposed to disengaging the instrumental system. If anxiety instead impaired the instrumental system, it would likely lead to increased exposure of harmful situations because, as highlighted above, the instrumental and Pavlovian systems are generally aligned to facilitate optimal behaviors in nature (Rangel et al., 2008). Based on these findings, we propose that the most plausible explanation for our results is that anxiety influences motivational behavior via modulation of aversive Pavlovian processes. However, we acknowledge that these results do not provide definitive evidence that the increased inhibition under threat is driven by aversive Pavlovian biases. In light of this, interpretations are tentative and future studies are warranted to confirm the present results.

As a further caveat, it should be noted that the no-go conditions were not significantly different from each other. We might expect performance to be significantly slower in the NGA condition compared with the NGW condition as the Pavlovian and instrumental controllers both promote inhibitory responses (slowing of responses) in the NGA condition while they compete in the NGW condition. However, the response bias, HR, and FAR scores all indicate that the reinforcement schedules were in fact successful in generating an action bias. This lack of difference might be attributed to RTs in the no-go conditions being at floor levels. Indeed, there was no significant difference between the GA condition under threat compared with the NGA condition under safe and threat. This is perhaps surprising as we would expect the NGA condition to produce the slowest responses as both Pavlovian and instrumental responses promote inhibition (while the slowing of responses in the GA condition under threat is only driven by the Pavlovian no-go bias). It thus seems that the slowest participants can perform on this task is capped at the level of the GA condition under threat. It is therefore not entirely clear whether the effect of ToS on Pavlovian-instrumental interactions is driven simply by an aversive Pavlovian bias, or if it is specific to when Pavlovian bias conflicts with instrumental processes in an aversive context. Another possibility might be that the conflict between Pavlovian and instrumental controllers in the NGW condition promote cautiousness and slowing, rendering it similarly slow as the NGA condition. A final possibility is that this specific task is simply insensitive to appetitive Pavlovian-instrumental conflict. However, we believe that if ToS affected appetitive conflict as well, it would still have been revealed by the present task. Specifically, if ToS induced a general slowing of responses, regardless of an appetitive or aversive context, we would also expect to see slowing of responses under threat in the GW condition. This is not supported by the data. If, on the other hand, ToS induced a greater reliance on Pavlovian appetitive biases or impaired instrumental control in the appetitive condition, we would expect *faster* responses under threat in the NGW condition. Faster response times would be possible, as evidenced by faster response times on other conditions. However, this effect of threat was not observed. Moreover, the present results are in line with previous studies demonstrating that ToS only affects aversive but not appetitive conditions (for a review see Robinson, Vytal, et al., 2013). In summary, our view is that the most parsimonious explanation for the results that were observed is that threat impacts aversive Pavlovian responding. However, future studies might plausibly explore the impact of stimulus presentation duration on RTs to attempt to tease these effects apart.

Finally, it should be noted that our main analysis slightly deviated from the original study (Crockett et al., 2009) such that the first practice block was chosen as the response latency baseline as opposed to the first neutral block. Prior to analysis, we reasoned that this would be the most appropriate measure of baseline RTs in our study. Another possible baseline could have been the first neutral block during the safe condition, in accordance with Crockett et al. (2009). However, due to the counterbalanced threat manipulation in our study, only half of the participants experienced the first neutral block under the safe condition. Power to detect an effect would therefore be reduced to ∼50% with this baseline, and perhaps more importantly, the threat manipulation would no longer be counterbalanced, rendering it an inappropriate baseline choice. Future studies should include a neutral block before the threat manipulation to assess baseline RTs, so as to replicate the analyses of Crockett et al. (2009) precisely.

In conclusion, this is the first study to suggest that ToS selectively promotes punishment-induced inhibition in motivational behavior. Importantly, the present study provides a potential mechanistic understanding of this: Adaptive anxiety promotes avoidant behavior in potentially harmful situations by increasing reliance on aversive Pavlovian processes.

## Supplementary Material

10.1037/xge0000363.supp

## Footnotes

1 It should be noted that the Pavlovian response in the face of punishments is generally situation-specific, such that fight reactions are elicited to proximal threats but inhibitory reactions are elicited to distal threats. Here, we focus on the latter but not the former Pavlovian behavioral response to potential losses.
