# Supplementary material for: Threat of Shock and Aversive Inhibition: Induced Anxiety Modulates Pavlovian-Instrumental Interactions
Source: J Exp Psychol Gen. 2017 Sep 14;146(12):1694–704. doi: 10.1037/xge0000363 (PMC5733814; doi:10.1037/xge0000363)
Supplement: Supplementary file 1 [file zfr999172966so1.docx]

**Supplemental Materials**

**Threat of Shock and Aversive Inhibition: Induced Anxiety Modulates Pavlovian-Instrumental Interactions**

**by A. Mkrtchian et al., 2017, *Journal of Experimental Psychology: General***

**http://dx.doi.org/10.1037/xge0000363**

***Reinforced go/no-go task***

*Raw reaction time (RT) analysis and results*

For purposes of full reporting, the raw RTs for correct go trials for all action-valence conditions in the threat and safe conditions were also analysed. The raw RTs were analysed with a repeated-measures ANOVA with threat (threat, safe), action bias (go, no-go) and valence (reward, punishment) as within-subjects factors.

Analysis of the raw RTs showed a main effect of action bias (*F*_(1,61)_=45.19, *p*<0.001, $\eta_{p}^{2}$=0.426), valence (*F*_(1,61)_=7.32, *p*=0.009, $\eta_{p}^{2}$=0.107), action bias by valence interaction (*F*_(1,61)_=9.61, *p*=0.003, $\eta_{p}^{2}$=0.136) as well as a significant threat by action bias by valence interaction *(F*_(1,61)_=5.26, *p*=0.025, $\eta_{p}^{2}$=0.079). This three-way interaction was driven by a significant action bias by valence interaction under threat (*F*_(1,61)_=12.00, *p*=0.001, $\eta_{p}^{2}$=0.164) but not under safe (*F*_(1,61)_=1.08, *p*=0.302, $\eta_{p}^{2}$=0.017). Under threat, participants were significantly slower to make a correct response to avoid punishment (GA) than to obtain a reward (GW) (*F*_(1,61)_=14.33, *p*<0.001, $\eta_{p}^{2}$=0.19) but there was no significant difference between NGW and NGA conditions (*F*_(1,61)_=1.09, *p*=0.302, $\eta_{p}^{2}$=0.017). The difference between threat and safe approached significance for the GA (*F*_(1,61)_=3.22, *p*=0.078, $\eta_{p}^{2}$=0.050), but not for GW (*F*_(1,61)_=0.062, *p*=0.804, $\eta_{p}^{2}$=0.001), NGW (*F*_(1,61)_=1.59, *p*=0.212, $\eta_{p}^{2}$=0.025), or NGA (*F*_(1,61)_=0.85, *p*=0.360, $\eta_{p}^{2}$=0.014) conditions.

Although the analysis of raw RTs differs slightly from the normalised RT results at the simple mains effects level, it is important to note that the raw RT analysis is a less sensitive method of assessing Pavlovian-instrumental interactions as it does not take into account baseline RTs, making it a noisier measure (Crockett et al., 2009). This analysis is therefore less well powered to assess Pavlovian-instrumental interactions and should therefore be interpreted with caution.

*Task order analysis*

We assessed task order by including it as a between-subjects factor but dropped it as it did not significantly affect any main or interaction effects (all *F*s<2.2, all *p*s>0.075).

*Performance on easy and hard trials in the reinforced go/no-go task*

**[Fig. S1]**

***SART task***

*Task order analysis*

To assess task order effects, two mixed ANOVAs were performed on the accuracy and RTs, respectively, with threat as within subjects factor and task order as a between-subjects factor. The effect of task order was not significant (all *F*s<1, all *p*s>0.6).

**Figures**


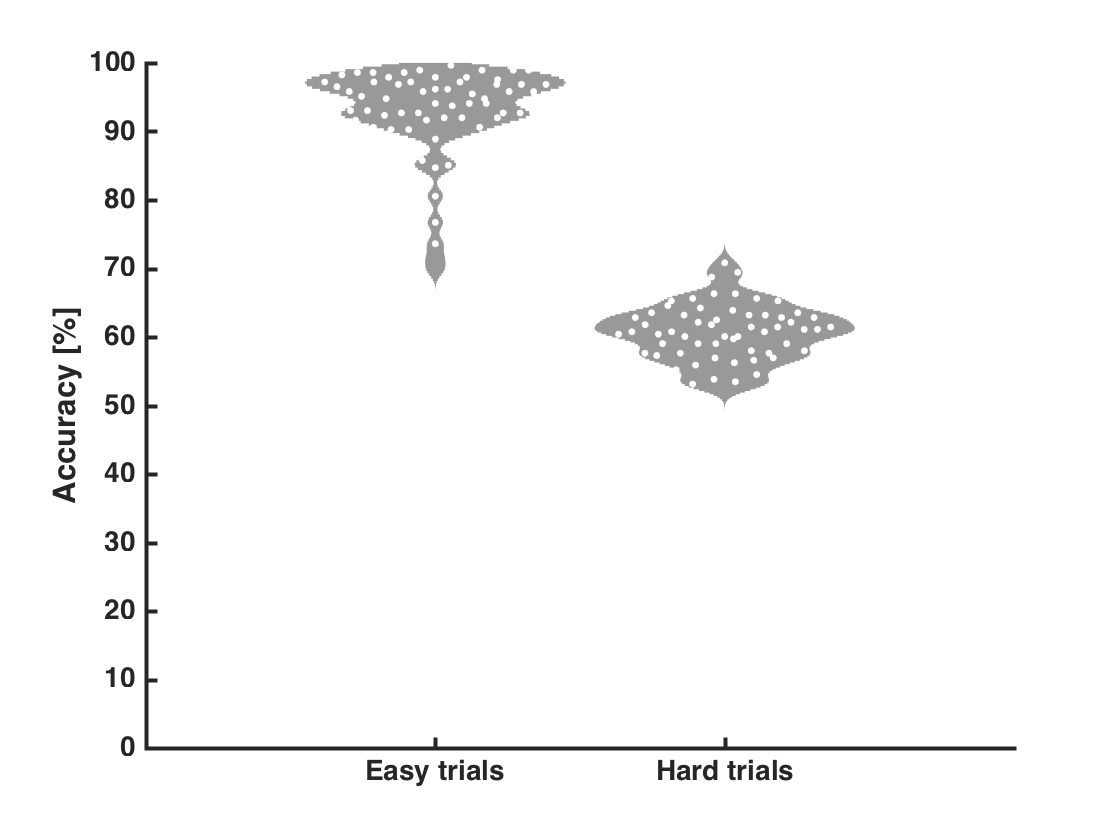

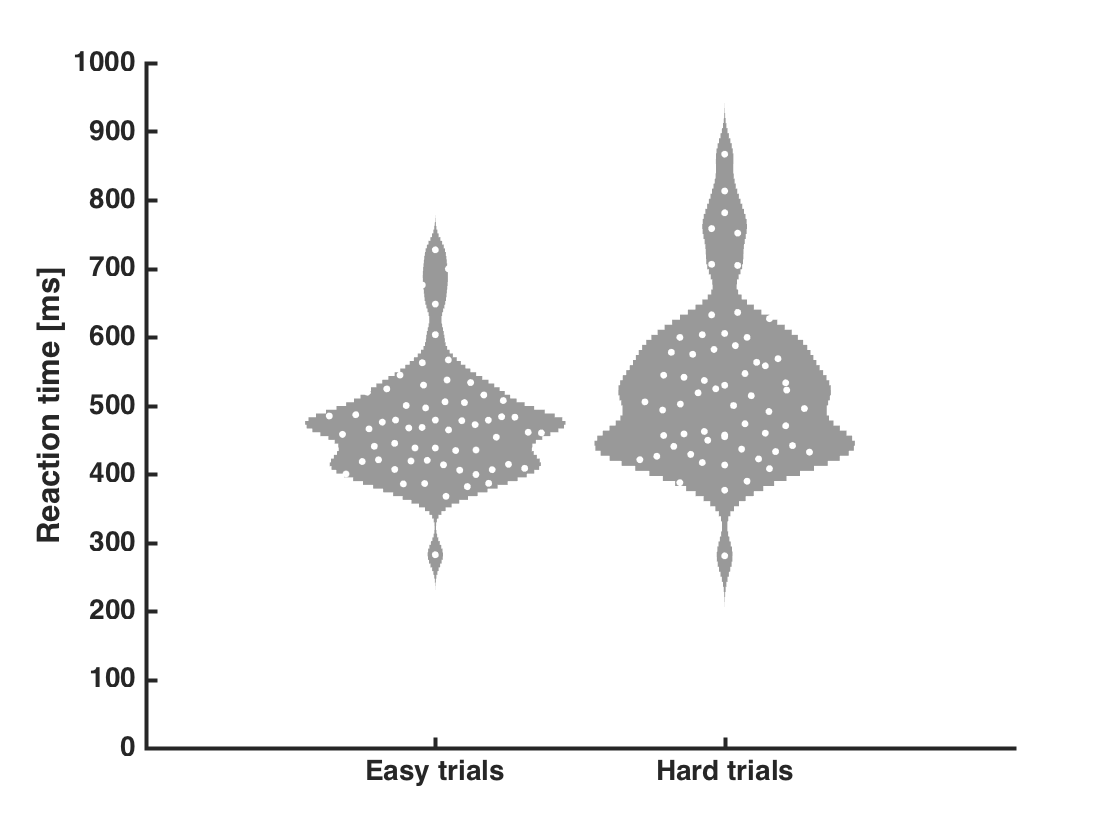


**a**

**b**

**Fig. S1:** Violin plots of performance on easy and hard trials in the reinforced go/no-go task. (a) Overall accuracy (across go and no-go trials) performance. (b) Overall reaction time performance on go trials. The data indicate that there is a ceiling effect on easy but not hard trials on the accuracy but not reaction time data.
